# Transglutaminase 2 moderates the expansion of mouse abdominal aortic aneurysms

**DOI:** 10.1016/j.jvssci.2021.04.002

**Published:** 2021-05-18

**Authors:** Kathryn J. Griffin, Kingsley R. Simpson, Cora M.L. Beckers, Laura M. Newell, Lih T. Cheah, Nadira Y. Yuldasheva, Siiri Iismaa, Christopher L. Jackson, Julian D.A. Scott, Richard J. Pease

**Affiliations:** a Leeds Institute of Cardiovascular and Metabolic Medicine, University of Leeds, Leeds, UK; b Bristol Heart Institute, University of Bristol, Bristol, UK; c Victor Chang Cardiac Research Institute, Darlinghurst, Australia

**Keywords:** Aneurysm, Transglutaminase 2, Factor XIII-A

## Abstract

**Objective:**

Previously published work has indicated that transcripts encoding transglutaminase 2 (TG2) increase markedly in a rat model of abdominal aortic aneurysm. This study determines whether TG2 and the related TG, factor XIII-A (FXIII-A), protect against aortic aneurysm development in mice.

**Methods:**

C57BL/6J wild-type, *Tgm2*^*–/–*^ knockout, *F13a1*^–/–^ knockout, and *Tgm2*^*–/–*^/*F13a1*^–/–^ double knockout mice were subjected to laparotomy and periaortic application of CaCl_2_.

**Results:**

*Tgm2*^*–/–*^ mice showed slightly greater aortic dilatation at 6 weeks after treatment when compared with wild type. However, vessels from *Tgm2*^*–/–*^ mice, but not wild-type mice, continued to dilate up to 6 months after injury and by 24 weeks, a greater number of *Tgm2*^*–/–*^ mice had developed aneurysms (16/17 vs 10/19; *P* = .008). Laparotomy resulted in a high death rate in *F13a1*^*–/–*^ knockout mice, more frequently from cardiac complications than from hemorrhage, but among *F13a1*^*–/–*^ mice that survived for 6 weeks after CaCl_2_ treatment, abdominal aortic aneurysm diameter was unaltered relative to wild-type mice. Laparotomy resulted in a higher death rate among *Tgm2*^*–/–*^/*F13a1*^*–/–*^ double knockout mice, owing to an increased frequency of delayed bleeding. Surprisingly, *Tgm2*^*–/–*^/*F13a1*^*–/–*^ double knockout mice showed a trend toward decreased dilatation of the aorta 6 weeks after injury, and this finding was replicated in *Tgm2*^*–/–*^/*F13a1*^*–/–*^ mice subjected to carotid artery injury. Levels of transcripts encoding TG2 were not increased in the aortas of injured wild-type or *F13a1*^*–/–*^ knockout mice relative to uninjured mice, although changes in the levels of other transcripts accorded with previous descriptions of the CaCl_2_ aneurysm model in mice.

**Conclusions:**

Knockout of *Tgm2*, but not *F13a1* exacerbates aortic dilatation, suggesting that TG2 confers protection. However, levels of TG2 messenger RNA are not acutely elevated after injury. FXIII-A plays a role in preventing postoperative damage after laparotomy, confirming previous reports that it prevents distal organ damage after trauma. TG2 promotes wound healing after surgery and, in its absence, the bleeding diathesis associated with FXIII-A deficiency is further exposed.

## Clinical relevance

There is a lack of medical treatments that inhibit the expansion of aneurysms and thus prevent or delay the need for surgical intervention. Transglutaminase 2 (TG2) has been proposed to play a role in the repair of tissues including blood vessels. Our results do not confirm that TG2 expression is acutely increased in response to aneurysm development, but instead suggest that absence of TG2 exacerbates experimental aneurysm development. Pharmacologic induction of TG2 expression may form part of a strategy to exploit the healing of aneurysmal arteries.

**Article Highlights**

• **Type of Research:** Basic science study
• **Key Findings:***Tgm2*^*–/–*^ mice show a small increase in aortic diameter 6 weeks after CaCl_2_ injury relative to control mice while vessels from *Tgm2*^*–/–*^ mice, but not wild-type mice, continued to dilate up to 24 weeks after injury. *Tgm2*^*–/–*^/*F13a1*^*–/–*^ double knockout mice show a trend toward decreased aortic dilatation and this finding was replicated in *Tgm2*^*–/–*^/*F13a1*^*–/–*^ mice subject to carotid artery injury.
• **Take Home Message:** Transglutaminase 2 (TG2) has been proposed to play a role in the repair of blood vessels. Our results do not confirm that TG2 expression is acutely increased after aneurysm development, but instead suggest that the absence of TG2 exacerbates experimental aneurysm development.

Arterial aneurysms exhibit thinning and disruption of the medial layer, resulting from the loss of elastin and vascular smooth muscle cells. Collagen synthesis may increase to stabilize aneurysms, but collagen itself undergoes accelerated degradation, leading to vessel dilatation and eventual rupture.^1^ Abdominal aortic aneurysms (AAA) are detected in the infrarenal aorta of 1.3% to 3.3% of males aged 65 to 80 years undergoing screening, with risk factors that include hypertension, atherosclerosis, and smoking, and account for 1% to 2% of deaths in this group.^1^ Both open and endovascular aneurysm repair carry significant risk^2^ and effective medical treatments that mitigate AAA development are not available currently.^3^

Targeting the proteases that degrade aortic proteins could potentially moderate aneurysm development. However, several proteases may need to be simultaneously inhibited to halt disease progression,^4^ and although absence of matrix metalloproteinase (MMP)9 inhibits aneurysm development in animal models,^5^, ^6^, ^7^ administration of the MMP inhibitor, doxycycline, to patients with small aneurysms failed to decrease aneurysm growth.^8^

Alternatively, it may be possible to promote the expression of enzymes that mediate arterial protection and repair. TGs comprise a family of eight enzymes that introduce N^ε^ (γ-glutamyl) lysine isopeptide cross-links between and within protein chains, conferring mechanical stability and proteolytic resistance.^9^ Among these, extracellular TG2^10^ cross-links matrix proteins including fibronectin^11^^,^^12^ and possibly isoforms of collagen.^11^ In addition, intracellular TG2 regulates gene expression and hence decisions of cell fate.^13^ Although many cells express TG2 basally, expression is induced in response to proinflammatory stimuli^14^ and when macrophages polarize to a reparative M2 phenotype.^15^ Accordingly, TG2 is present in injured tissues, such as the vulnerable shoulder regions of human atherosclerotic plaques.^16^^,^^17^

A second TG, blood clotting factor XIII-A (FXIII-A), circulates in plasma as the heterotetramer FXIII-A_2_B_2_ and is present within the cytosol of cell types including megakaryocytes, platelets, and macrophages as the homodimer FXIII-A_2_.^18^ Plasma FXIII-A_2_B_2_ cross-links matrix proteins including fibrin, fibronectin, collagen, and vitronectin^19^ and contributes to placental maintenance^20^ and dermal repair.^21^ Macrophage FXIII-A expression is induced upon differentiation to a reparative M2 phenotype^22^ and cooperates with plasma FXIII-A_2_B_2_ to stabilize myocardial scars^23^ and with macrophage-derived TG2 to facilitate inward arterial remodelling.^24^

Munezane et al^25^ reported that TG2 expression increases when infrarenal aortic aneurysms are induced in rats. We previously observed that lack of TG2 lessens the resistance of carotid arteries to the mechanical strain of ligation.^26^ Given that weakened vessels are prone to aneurysm development,^27^ this finding might imply that TG2 would confer protection. We are unaware of evidence that FXIII-A directly influences aneurysm development, although various studies have proposed that FXIII-A overlaps in function with TG2 (eg,^28^). Therefore, to address the roles of TG2 and FXIII-A in aneurysm development, we have induced aneurysms in the infrarenal aorta of*Tgm2*^*–/–*^ knockout, *F13a1*^*–/–*^ knockout, and *Tgm2*^*–/–*^*/F13a1*^*–/–*^ double knockout mice.

## Methods

Animal housing, husbandry, and procedures were conducted in accordance with guidelines and regulations of the University of Leeds and of the United Kingdom Home Office. Mice had ad libitum access to water and to a standard chow diet (softened for 24 to 48 hours after surgery).

### Generation of TG-deficient mice

The breeding and genotyping of *Tgm2*^*+/–*^ mice^29^ and of *F13a1*^–/–^ mice and *Tgm2*^–/–^/*F13a1*^*–/–*^ mice each back-crossed onto a more than 97.5% C57BL/6J background^26^ have been described previously. Equal numbers of male and female mice at 8 to 10 weeks of age were used for each procedure.

### Harvesting and biochemical analysis of arteries

Mice were anaesthetized with isoflurane and then subjected to perfusion exsanguination via the left ventricle with 5 mL phosphate-buffered saline (Sigma Aldrich, Dorset, UK). The descending aorta to the iliac bifurcation was snap frozen in liquid N_2_, stored at –80°C, and either (i) processed to determine messenger RNA (mRNA) levels^26^ or (ii) dehydrated to constant weight under vacuum at 80°C, lysed for 48 hours at 4°C in 750 μL of Na_2_HPO_4_ (50 mmol.L^−1^), NaCl (50 mmol.L^−1^), 1% Triton X-100, 0.1% SDS, pH 7.4, and subjected to centrifugation (14,000×*g* for 10 minutes at 4°C). The supernatant fraction was assayed to determine protein (bicinchoninic acid assay; Sigma-Aldrich) and lactate dehydrogenase activity (CytoTox 96 lactate dehydrogenase assay; Promega, Madison, Wisc). DNA was assayed after forming a fluorescent complex for 2 hours with Sybr green I gel stain (1:20,000 dilution of stock solution [ThermoFisher Scientific, Waltham, Mass], in 50 mmol.L^−1^ Na_2_HPO_4_, 2 mol.L^−1^ NaCl, pH 7.4, λ_ex_ = 493 nm, λ_em_ = 530 nm). Pellets were used to measure oxalic acid-soluble elastin or pepsin-soluble collagen (Fastin or Sircol assays, respectively; Biocolor, Carrickfergus, County Antrim, UK).

### Wire myography

Abdominal aortas (n = 4) were dissected in cold Hanks buffered salt solution^30^ and two rings from each (1 mm in length) were mounted in a myograph (610 mol/L; Danish Myograph Technology, Hinnerup, Denmark), equilibrated for 30 minutes, and then placed under normalized tension in Krebs–Henseleit buffer gassed with 5% CO_2_/95% O_2_ at 37°C.^31^ The contractile responses to KCl (60 mmol.L^−1^) and to phenylephrine (1 μmol.L^−1^) were verified, after which the response to increasing concentrations of phenylephrine was determined. The median effective concentration values were estimated using Origin software (OriginLab Corporation, Northampton, Mass). Finally, endothelial integrity was confirmed by showing that carbachol (1 μmol.L^−1^) induced relaxation of vessels that had contracted in response to phenylephrine (1 μmol.L^−1^).

### Aneurysm induction

Mice were anaesthetized and then either the abdominal aorta was exposed or the right common carotid artery was exposed and isolated from surrounding tissue using a silicone strip (Eddingtons Ltd, Hungerford, UK)*.* Subsequently CaCl_2_ (0.5 mol.L^−1^) or NaCl (0.15 mol.L^−1^) was applied to the artery (2 × 7 minutes), and the exposed area was rinsed with NaCl (0.15 mol.L^−1^). The mice received an intraperitoneal injection of Buprenorphine 0.1 to 1.0 mg/kg (Vetergesic, Reckitt Benckiser, Slough, UK) before recovery. Arteries were imaged in situ before CaCl_2_ treatment and at termination (6 weeks or 24 weeks) using the OPMI-PICO video micrometer (Carl Zeiss AG, Jena, Germany). Vessel measurements were independently determined by two investigators using Image-Pro software (MediaCybernetics, Rockville, Md).

### Histology

Arteries for histologic examination were perfusion-fixed in 4% paraformaldehyde in 50 mmol.L^−1^ Na_2_HPO_4_, 150 mmol.L^−1^ NaCl, pH 7.4. Serial transverse sections were cut at 5-μm intervals from the left renal artery to iliac bifurcation and stained with Miller's elastic Van Gieson, hematoxylin and eosin, picrosirius red, or alizarin red S and were imaged using an Olympus BX61WI inverted microscope with an XC10-IR camera under the control of CellSens software (Olympus, Tokyo, Japan). Fibrillar collagen density adjacent to damaged and undamaged regions from Van Gieson stained aortic sections was determined by excitation at 800 nm with a Chameleon laser and collecting the second harmonic signal (400 nm) through a 10× objective lens and an EF SP 485 IR++ filter onto a Zeiss LSM NDD R2 detector using a Zeiss 710 multiphoton microscope and analyzing 10 × 10 pixel square regions using ImageJ.

All image analysis was carried out in a blinded manner.

### Quantification of messenger RNA

Mouse aortas (n = 8-10) were disrupted in TRIzol (ThermoFisher Scientific) using a TissueLyser II (Qiagen, Hilden, Germany), The nuclei acid fraction was precipitated from the aqueous layer and mRNA processed for reverse transcriptase polymerase chain reaction, as previously described,^26^ using the primer pairs shown in the Supplementary Table (online only). Transcript levels were normalized to those encoding ribosomal protein subunit-32, using the 2^–ΔCt^ method.^32^

### Immunohistochemistry

Immunofluorescent detection of CD163 and FXIII-A antigens in sections of mouse aorta was carried out as previously described.^33^

### Statistical analysis

Unless stated, all are is presented as mean ± standard deviation. All values were analyzed using Prism 7 (GraphPad software, San Diego, Calif). Comparisons between each group were carried out using the unpaired Student *t* test (with Welch's correction) or one-way analysis of variance (with Bonferroni correction) for multiple groups. A Kruskal-Wallis test with Dunn's multiple comparison test was used for any data that were not normally distributed; normality was assessed using Shapiro-Wilk testing. Contingency data (proportion of mice developing an aneurysm; defined as increase in diameter of >50%) was analyzed using Fisher's exact test of proportions. Significance was accepted where the *P* was less than .05.

## Results

### Baseline properties of mouse aortas are similar between genotypes

**Fig 1:**
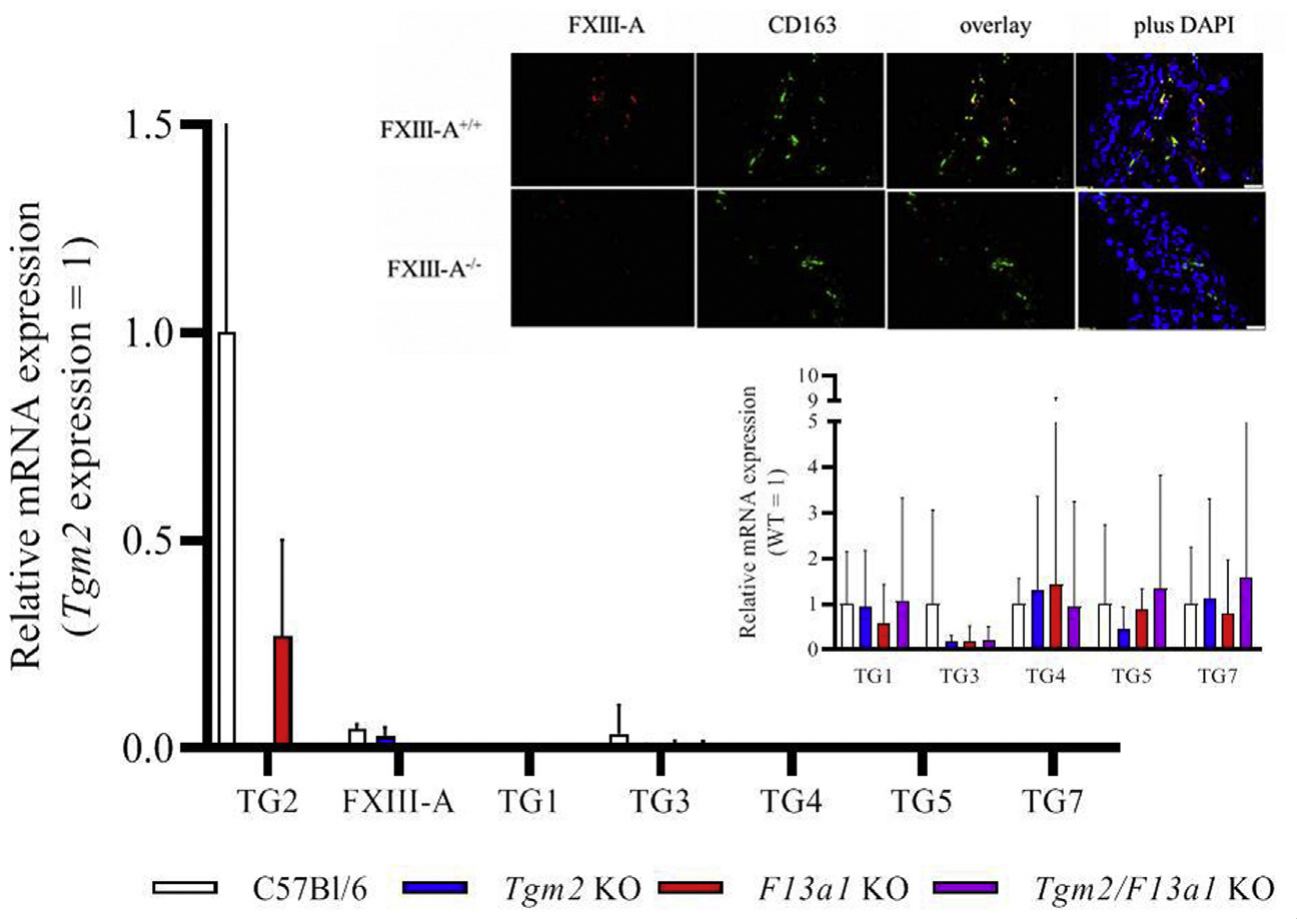
Transglutaminase (*TG*) expression in aortas from C57Bl/6J wild-type mice and transglutaminase knockout mice. Transcripts encoding TG2 and factor XIII-A (*FXIII-A*) were detected in C67Bl/6J wild-type (WT) mice but were absent from the relevant knockout mice. Transcripts encoding TG3 were detectable in WT mice but did not increase in knockout mice. Transcripts encoding TG1, TG4, TG5, and TG7 were present in WT mouse aortas at very low concentrations relative to TG2 expression (*main graph*) and did not increase in *Tgm2*^*–/–*^ or *F13a1*^*–/–*^ knockout mice (*lower inset*). FXIII-A protein was present in CD163 positive cells (macrophages) in WT mice, but was not detected in CD163 macrophages in *F13a1*^–/–^ knockout mice (*upper inset*). *mRNA,* Messenger RNA.

The mRNAs encoding TG2, FXIII-A. and TG3 were the most abundant TG transcripts in the wild-type aorta. Other TG mRNAs were present at very low levels or were undetectable (TG6). Knockout of the *F13a1* gene, the *Tgm2* gene, or both did not result in induction of other *Tgm* genes to a level expected to compensate for either gene knockout. Immunofluorescence confirmed that FXIII-A was present in CD163 positive macrophages of aorta (Fig 1), as previously described in skin^34^ and heart.^33^

**Fig 2:**
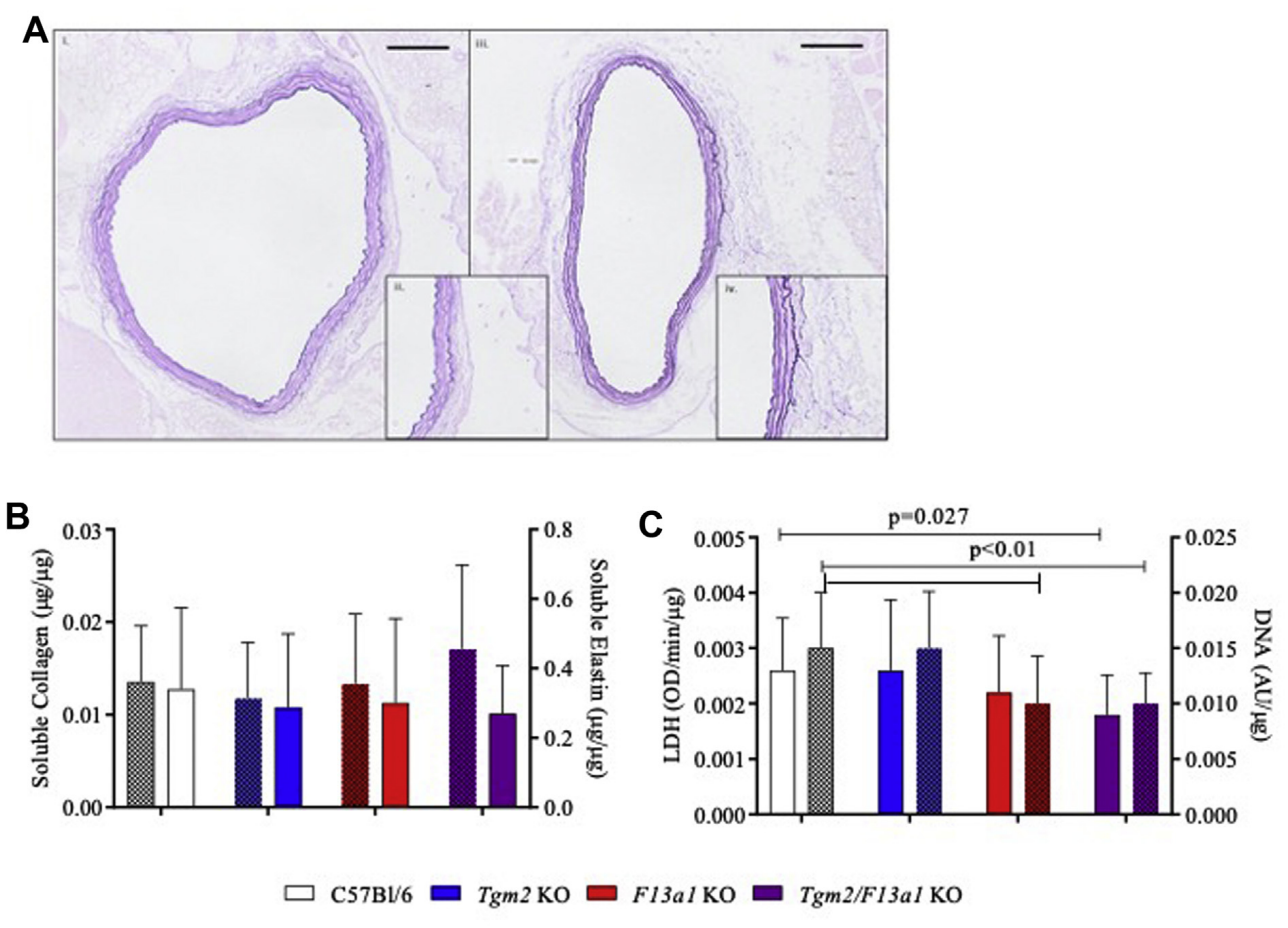
Mice lacking factor XIII-A (FXIII-A) have decreased aortic cellularity but similar elastic morphology. **A,** Representative Miller's elastic Van Gieson stained sections (5 μm) from a basal (unoperated) aorta from (i and ii) a C57Bl/6J wild-type mouse and (iii and iv) a *Tgm2*^*–/–*^*/F13a1*^*–/–*^ mouse. Insets to show lamellar structure; scale bar represents 100 μm. **B,** Concentrations of oxalate-soluble elastin (*plain bars*) and of pepsin-soluble collagen (*patterned ba*rs) expressed per microgram of SDS-soluble protein in C57Bl/6J and transglutaminase (TG) knockout mouse aortas. Neither protein varied in concentration between mice of different genotypes (n = 8-12). **C,** Activity of lactate dehydrogenase (*LDH*) (*plain bars*) and concentration of DNA (*patterned bars*) in the aorta (n = 25-28). Both LDH and DNA content were decreased in *Tgm2*^*–/–*^*/F13a1*^*–/–*^ mice and DNA content was decreased in *F13a1*^*–/–*^ knockout mice.

The aortic structure was similar between genotypes, illustrated by representative images of wild-type and *Tgm2*^*–/–*^*/F13a1*^*–/–*^ aortas (Fig 2, *A*). The mean lamellar number (n = 4) did not differ between genotypes (C57Bl/6J, 4.76 ± 0.54; *Tgm2*^*–/–*^, 4.38 ± 0.49; *F13a1*^*–/–*^, 4.60 ± 0.49; *Tgm2*^*–/–*^*/F13a1*^*–/–*^, 4.64 ± 0.54). Measurements of elastin were consistent with published values^35^^,^^36^ and did not vary between genotypes. Pepsin-soluble collagen was measured by the Biocolor assay, which preferentially detects newly synthesized collagen, and showed no statistically significant difference between groups (Fig 2, *B*). Elastin and collagen could not be assayed in the same sample, precluding estimation of errors in the ratio. However the ratio (average (soluble collagen/protein)/(average [soluble elastin/protein]) seemed to be higher in *Tgm2*^*–/–*^*/F13a1*^*–/–*^ mice (0.0633) than in other genotypes (C57Bl/6J, 0.0398; *Tgm2*^*–/–*^, 0.0458; *F13a1*^*–/–*^, 0.0455 [n = 10-12]). DNA content and lactate dehydrogenase activity, measured as indices of cellularity, were decreased in *F13a1*^*–/–*^ and *Tgm2*^*–/–*^*/F13a1*^*–/–*^ aortas (Fig 2, *C*).

**Fig 3:**
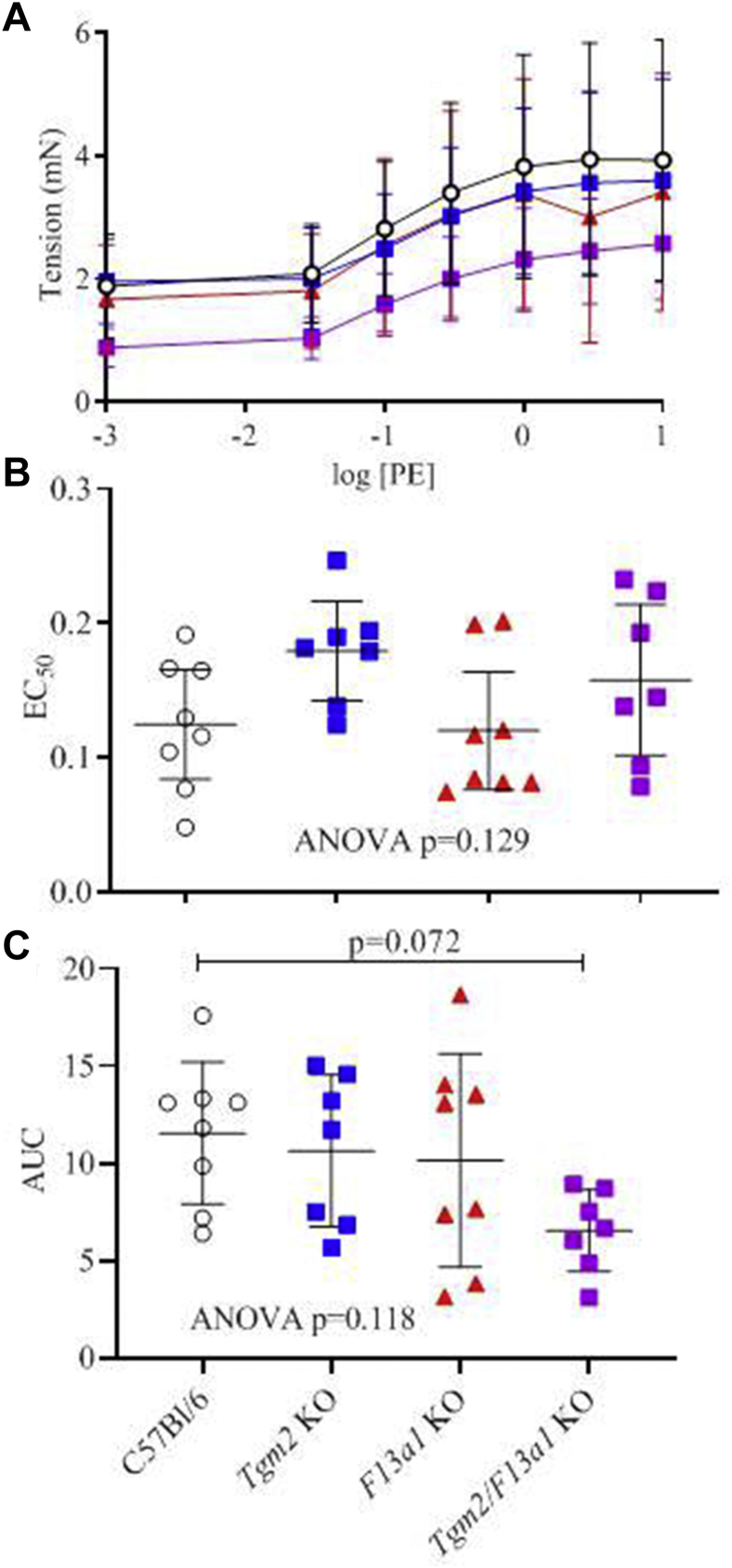
Ex vivo function of aortas from wild-type and transglutaminase (TG) knockout mice. **A,** Increase in force exerted by excised aortas in response to increasing concentrations of phenylephrine (*PE*). **B,** Calculated concentrations (median effective concentration [*EC*_*50*_]) at which contraction generated half maximal force. **C,** Area under the curves (*AUC*) from experiments in (**A)**. There was a tendency for contraction to be weaker in *Tgm2*^*–/–*^*/FXIII-A*^*–/–*^ mice at all concentrations of PE, resulting in a lower AUC. *ANOVA,* Analysis of variance; *KO,* Knockout.

The basal tension exerted by excised aortas from *Tgm2*^*–/–*^*/F13a1*^*–/–*^ mice tended to be lower than that exerted by wild-type, *Tgm2*^*–/–*^ or *F13a1*^*–/–*^ mice (*P* = .072). The median effective concentration for the phenylephrine response and the maximal increase in aortic tension were similar between genotypes (Fig 3). In summary, while the properties of aortas from *F13a1*^*–/–*^ and *Tgm2*^*–/–*^ single knockout mice appeared similar to C57Bl/6J wild-type mice, there were detectable differences in the basal state of *Tgm2*^*–/–*^*/F13a1*^*–/–*^ double knockout aortas that could affect their response to aneurysm development.

### Laparotomy is followed by failure to thrive and high mortality in mice lacking FXIII-A

**Fig 4:**
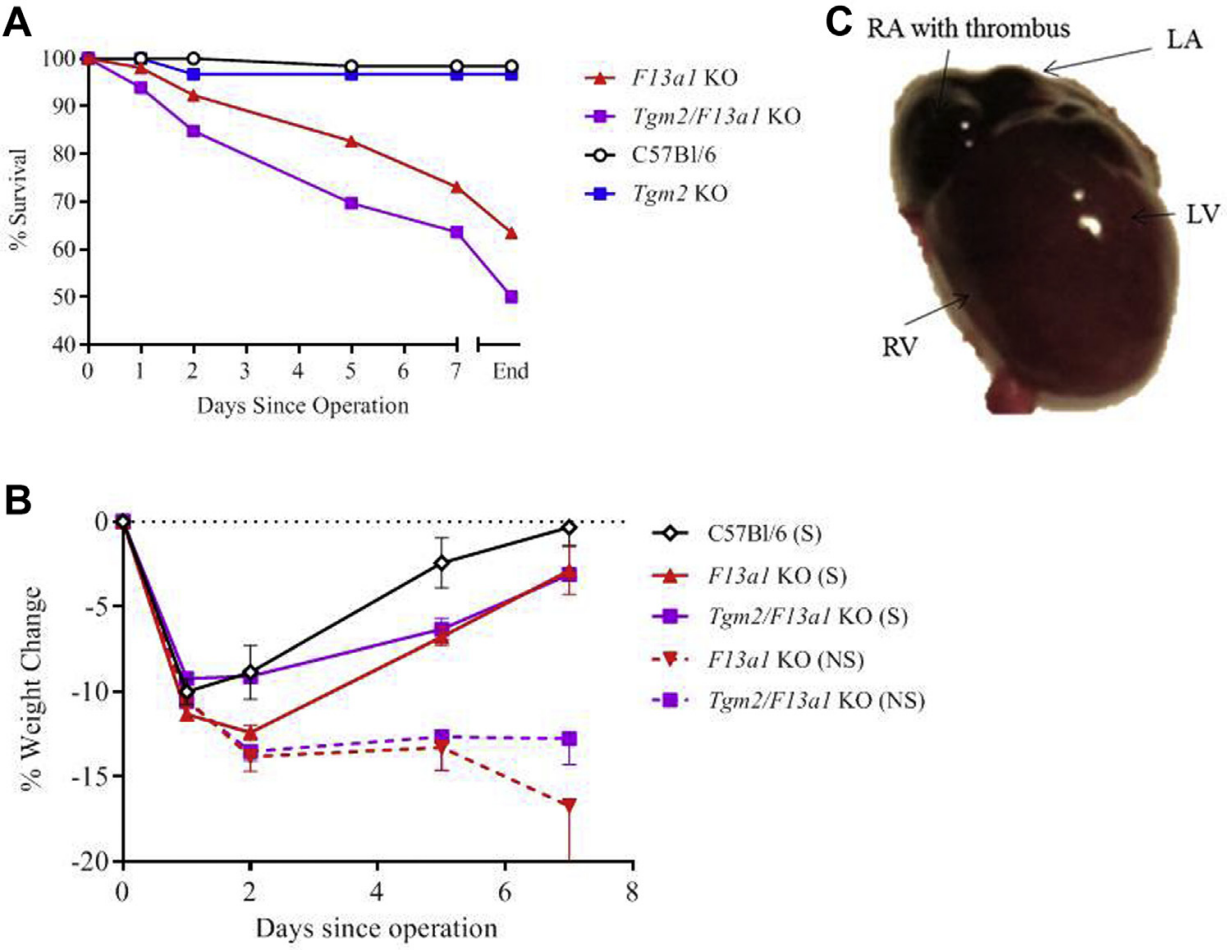
Mice lacking factor XIII-A (FXIII-A) show increased mortality after laparotomy. **A,***F13a1*^*–/–*^ knockout (*KO*) and *Tgm2*^*–/–*^*/F13a1*^*–/–*^ double knockout mice showed lower survival after laparotomy than C57Bl/6J wild-type mice. *End* represents scheduled termination date at 6 weeks. **B,** The average change in body weight of mice after laparotomy. Mice that would survive (*S*) regained weight to within 5% of baseline by 7 days. Mice that would not survive for 6 weeks after the operation (*NS*) showed little or no weight gain. **C,** Cardiac abnormalities present in a representative *F13a1*^–/–^ mouse after laparotomy, including a dusky myocardium. Upon opening, thrombus was detected within the right atrial cavity (not shown). *LA,* Left atrium; *LV,* left ventricle; *RA,* right atrium; *RV,* right ventricle.

Despite recovering from surgery, a high proportion of *F13a1*^*–/–*^ mice failed to regain weight and died, often within 1-5 days (Fig 4, *A*, *B*). Necropsy frequently showed blackened, thrombus-filled atria (Fig 4, *C*) and/or multiple discrete areas of focal necrosis in the bowel, spleen, and liver. Postoperative mortality was further increased in *Tgm2*^*–/–*^*/F13a1*^*–/–*^ mice over *F13a1*^*–/–*^ mice (Table) and the increase was largely attributable to delayed bleeding. A comparable death rate among NaCl-treated (sham-operated) *Tgm2*^*–/–*^*/F13a1*^*–/–*^ mice indicated that deaths were not a consequence of aneurysm development.

**Table:** Mortality and aneurysm development rate in mice following periaortic application of CaCl2 (0.5 mol.L^−1^ or NaCl (0.15 mol.L^−1^)

Genotype		Overall operative mortality (%)	Causes of death (% of deaths within group)	Aneurysm prevalence at 6 -weeks after injury	Aneurysm prevalence at 24 weeks after injury
C57Bl/6J	NaCl	9.4^a^		-	-
	CaCl_2_			17/28 (60.7%)	10/19 (52.6%)
*Tgm2*^*–/–*^	NaCl	3.4		-	-
	CaCl_2_			20/27 (74.1%)	17/17 (94.1%)
*F13a1*^*–/–*^	NaCl	37.3	Bleeding 11% Cardiac +/- thrombotic 37%	-	-
	CaCl_2_			16/26 (61.5%)	-
*Tgm2*^*–/–/*^*F13a1*^*–/–*^	NaCl	62.0	Bleeding 27% Cardiac +/- thrombotic 27%	-	-
	CaCl_2_			7/19 (36.8%)	-

Values are number/total number (%) unless otherwise indicated.

Aneurysm prevalence (absolute numbers and percentage) of mice developing an aneurysm (defined as aortic dilatation >50%) in each genotype group. Vessel dilatation was defined at the time of harvest at either 6 or 24 weeks after CaCl_2_ injury.

a The mortality of wild-type mice undergoing CaCl_2_ injury or NaCl sham procedure includes the first mice operated upon during familiarization with the method. The mortality rate among *Tgm2*^*–/–*^ mice (3.4%) more accurately reflects the incidental death rate among wild-type mice post-familiarization. Deaths post-familiarization were very high in *F13a1*^*–/–*^ mice and *Tgm2*^*–/–*^*/F13a1*^*–/–*^ mice. Other causes of death include fighting post-operation, bowel ischemia and wound complications. A large renal mass was detected in one *Tgm2*^*–/–*^*/F13a1*^*–/–*^ mouse. In some cases, the cause of death was unknown.

### Knockout of *Tgm2* exacerbates aneurysm progression, but FXIII-A does not exhibit functional redundancy with TG2

**Fig 5:**
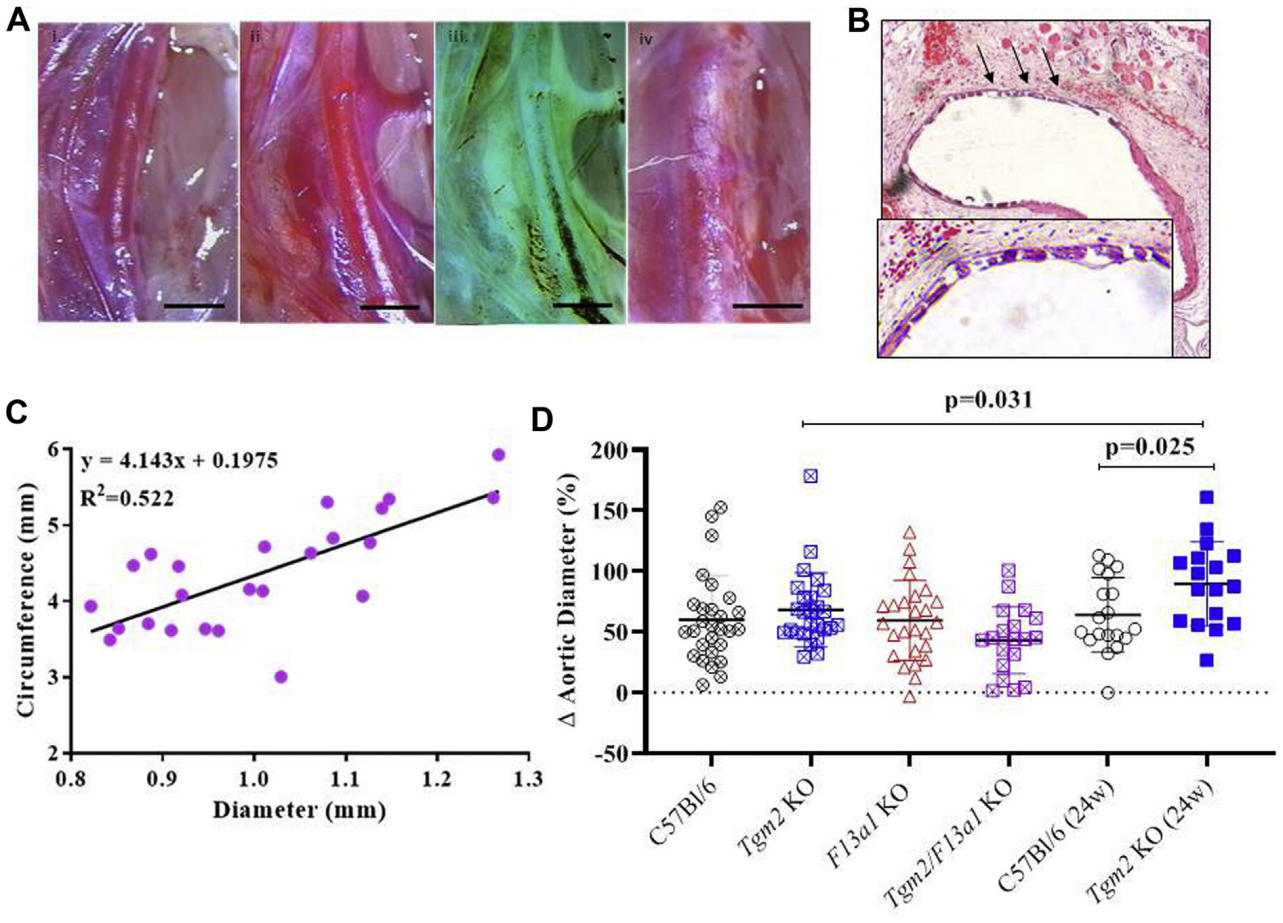
**A,** Representative images of aortas from wild-type mice captured: (i) before treatment; (ii) 6 weeks after the application of 0.15 mol.L^−1^ NaCl, native image; (iii) contrast inversion of image (ii); and (iv) 6 weeks after the application of 0.5 mol.L^−1^ CaCl_2_. Scale bars represent 1 mm. Treatment with CaCl_2_, but not NaCl, induced surface inflammation and focal dilatation. Contrast inversion afforded better resolution of the vessel margins and facilitated the measurement of the external diameter. **B,** Transverse section (stained with hematoxylin and eosin and imaged at 10× original magnification) of a C57Bl/6J wild-type aorta obtained 6 weeks after exposure to 0.5 mol.L^−1^ CaCl_2_. The ventral face (marked with *arrows* and shown in the *inset*) displays elastin breakage and fiber loss. **C,** The external diameter of CaCl_2_-treated arteries from wild-type mice is linearly related to the vessel circumference, confirming luminal dilatation. **D,** The percentage change in aortic diameter from baseline (mean ±95% confidence intervals) is shown at 6 weeks (*crossed symbol*) and 24 weeks (24 weeks, *filled symbol*) after treatment with 0.5 mol.L^−1^ CaCl_2_. There was no statistically significant difference between genotypes at 6 weeks of age (analysis of variance; *P* = .097), although there was a tendency for dilatation to be less in *Tgm2*^*–/–*^*/F13a1*^*–/–*^ double knockout mice. At 24 weeks, the mean aortic diameter had further increased in the *Tgm2*^*–/–*^ mice (6 weeks vs 24 weeks; *P* = .025), causing the average diameter to be greater than that in the C57Bl/6J wild-type mice (*P* = .031). *KO,* Knockout.

Exposure to CaCl_2_ caused surface inflammation and an increase in external aortic diameter in all genotypes (Fig 5, *A*). Transverse sections showed elastic fiber breakage (Fig 5, *B*) and were used to measure internal vessel circumference. Regression analysis indicated that the ratio Δ_(internal circumference)_/Δ_(external diameter)_ was 4.1 (Fig 5, *C*), which confirmed that luminal dilatation contributed to the increase in external diameter (expected value = π). Although a regression analysis cannot exclude that wall thickening also contributed to the increase in external diameter, this finding was not apparent by visual inspection of the sections, whereas wall thickening in the absence of dilatation would have generated a line of zero gradient. *Tgm2*^*–/–*^ mice showed slightly greater aortic dilatation 6 weeks after treatment (67.5 ± 30.2%) than wild-type mice (60.0 ± 36.3%; *P* = .097) (Fig 5, *D*). Aneurysms further progressed in *Tgm2*^*–/–*^ mice in the period up to 24 weeks (89.5 ± 34.5% dilatation [*P* = .025] relative to *Tgm2*^*–/–*^ mice at 6 weeks), but showed minimal progression in wild-type mice (64.1 ± 30.2% dilatation at 24 weeks [*P* = .57] relative to wild-type at 6 weeks). Defining aneurysms as a 50% increase in aortic diameter,^37^ 16 of the 17 *Tgm2*^*–/–*^ mice developed aneurysms at 24 weeks as opposed to 10 of the 19 wild-type mice (*P* = .008).

**Fig 6:**
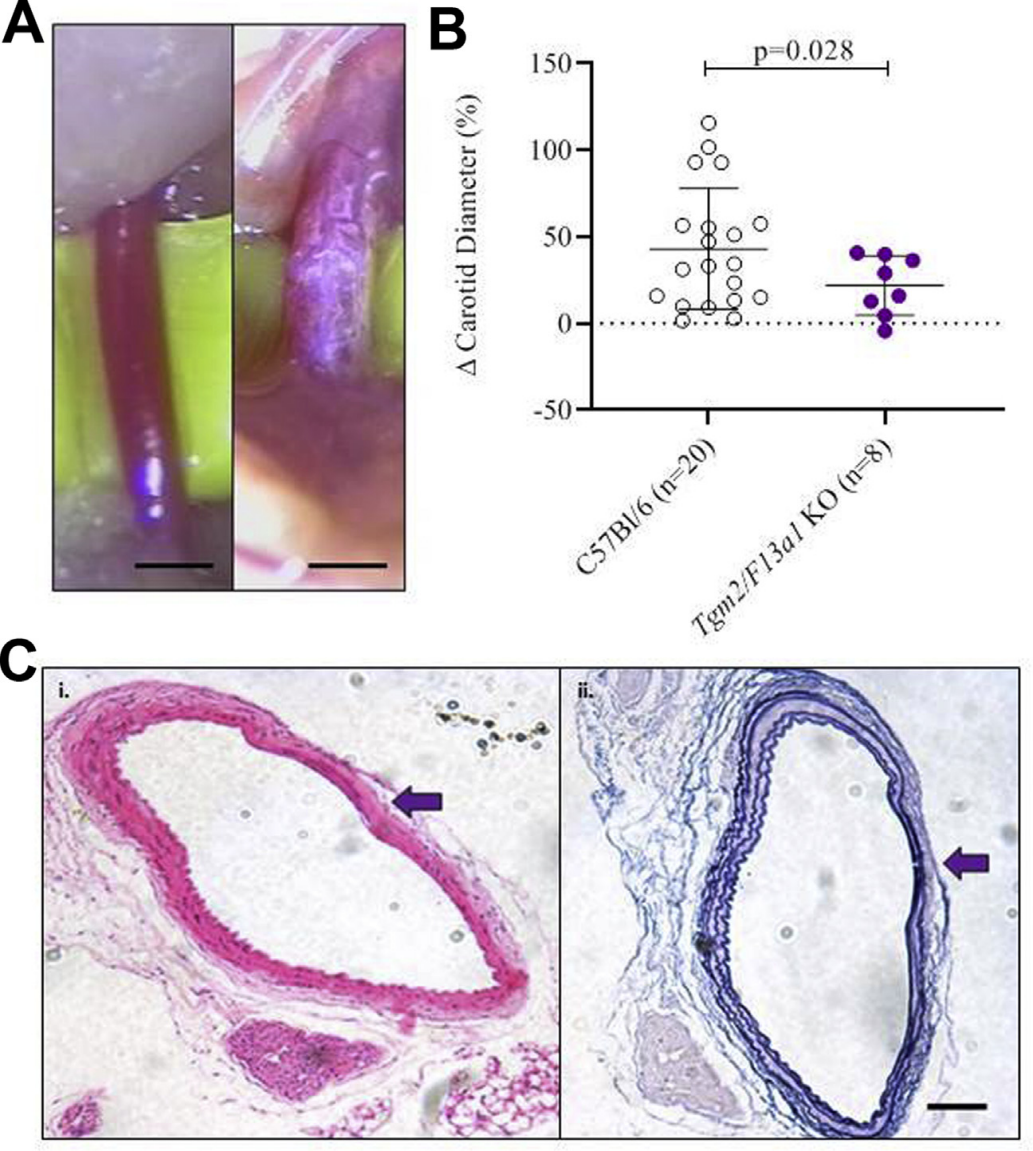
*Tgm2*^*–/–*^*/F13a1*^*–/–*^ mice show decreased dilatation of the carotid artery after application of CaCl_2_. **A,** Representative images of the carotid injury model of a C57Bl/6J wild-type mouse before *(left)* and after *(right)* treatment with 0.5 mol.L^−1^ CaCl_2_. Scale bars represent 500 μm. **B,** The percent change from baseline in the external diameter of carotid artery 6 weeks after treatment with 0.5 mol.L^−1^ CaCl_2_ is greater in C57Bl/6J wild-type than in *Tgm2*^*–/–*^*/F13a1*^*–/–*^ double knockout (*KO*) mice (*P* = .028). **C,** Representative transverse sections (5 μm) of a C57Bl/6J wild-type carotid artery 6 weeks after application of 0.5 mol.L^−1^ CaCl_2_, and stained with (i) hematoxylin and eosin stain and (ii) Miller's elastic Van Gieson stain. Elastic flattening and breakage were apparent in wild-type and *Tgm2*^*–/–*^*/F13a1*^*–/–*^ (not shown) carotid arteries following injury. Scale bar represents 100 μm.

In view of the possibility that FXIII-A might act redundantly with TG2 to influence aneurysm development, aortic dilatation was also measured 6 weeks after CaCl_2_ treatment in *F13a1*^*–/–*^ mice and *Tgm2*^*–/–*^*/F13a1*^-/-^ mice. Dilatation was essentially equal in *F13a1*^*–/–*^ mice (59.4 ± 32.9%) and wild-type mice, but tended to be less in *Tgm2*^*–/–*^*/F13a1*^–/–^ mice (43.1 ± 27.5%; *P* = .097). Although the cause of death after laparotomy in *Tgm2*^*–/–*^*/F13a1*^*–/–*^ mice seemed to be unrelated to aneurysm development, it seemed possible that *Tgm2*^*–/–*^*/F13a1*^*–/–*^ mice that would otherwise have developed a large AAA were more likely to die prematurely than those developing a small aneurysm. To address this phenomenon, we also induced aneurysms in the carotid artery and all mice survived 6 weeks after CaCl_2_ treatment. Similar to the abdominal aorta, dilatation of the carotid artery was less in *Tgm2*^*–/–*^*/F13a1*^*–/–*^ mice (20.7 ± 15.9%) than wild-type mice (43.1 ± 34.8%; *P* = .028) (Fig 6), suggesting that the decrease in aortic dilatation in *Tgm2*^*–/–*^*/F13a1*^*–/–*^ mice was not biased by mortality. Although the basis for this unexpected decrease in dilatation is unclear, the results exclude the possibility that FXIII-A acts redundantly with TG2 to inhibit aneurysm protection.

In view of the high mortality associated with laparotomy in mice lacking FXIII-A, and the lack of evidence that FXIII-A protects against aneurysm development, aneurysm development at 24 weeks was not assessed in either *F13a1*^*–/–*^ or *Tgm2*^*–/–*^*/F13a1*^*–/–*^ mice.

### Collagen density is decreased adjacent to areas of elastic breakage and is decreased in *Tgm2*^*–/–*^mice relative to wild-type mice

**Fig 7:**
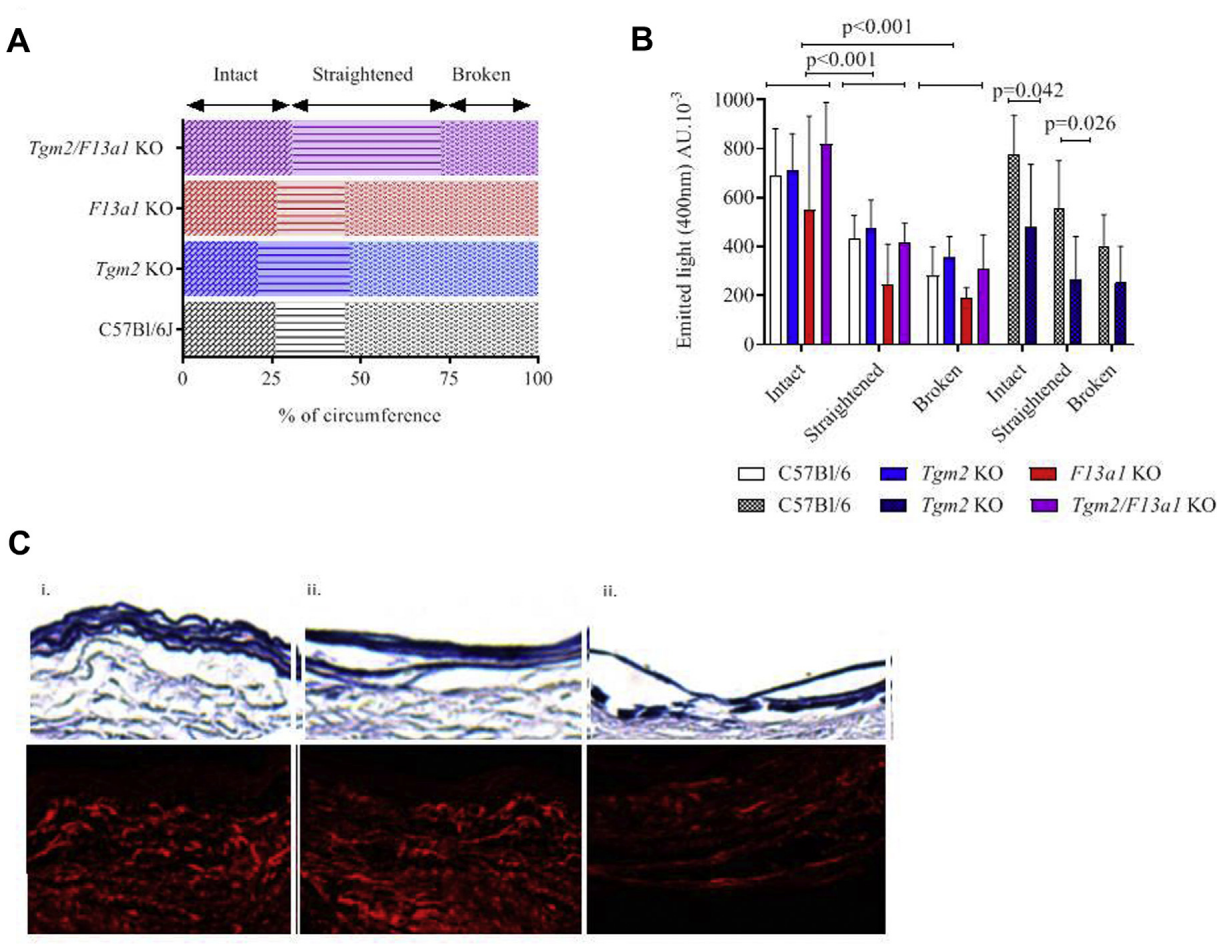
Collagen density is decreased adjacent to areas of medial damage. **A,** There is no significant difference between genotypes in the percentage distribution of regions of apparently healthy media, straightened elastic lamina, or broken elastic lamina in aortas examined 6 weeks after CaCl_2_ treatment. **B,** Representative Miller's elastic Van Gieson stained images of the media (*above*) and second harmonic images of fibrillar collagen in the adjacent adventitia (*below*) in areas in which the lamina appeared: (i) healthy, (ii) straightened or (iii) broken. **C,** The density of fibrillar collagen, measured in arbitrary units (AU) by second harmonic signal at 400 nm decreased adjacent to areas of laminar damage in mice of all genotypes, but there is no difference between genotypes 6 weeks after CaCl_2_ treatment (*plain bars*). However, when measured 24 weeks after CaCl_2_ treatment, the collagen density is significantly reduced in *Tgm2* knockout (*KO*) mice relative to wild-type mice, adjacent to areas of healthy and straightened elastin (*patterned bars*).

The fractional areas within aneurysms of wavy elastin (normal), straightened elastin (damaged), or broken elastin 6 weeks after CaCl_2_ treatment did not differ significantly between genotypes (Fig 7, *A*). However, elastin breakage tended to be decreased in *Tgm2*^*–/–*^*/F13a1*^*–/–*^ mice in accord with their reduced dilatation. In each genotype, the density of fibrillar collagen was decreased adjacent to regions of elastic fiber flattening and was further decreased adjacent to regions of elastic fiber breakage (Fig 7, *B*, *C*). Fibrillar collagen density at regions of similar damage did not differ between genotypes after 6 weeks, but was decreased adjacent to intact and flattened regions in 24-week aneurysms in *Tgm2*^*–/–*^ mice (relative to wild-type mice), consistent with the increased aneurysm size seen at this time point.

**Fig 8:**
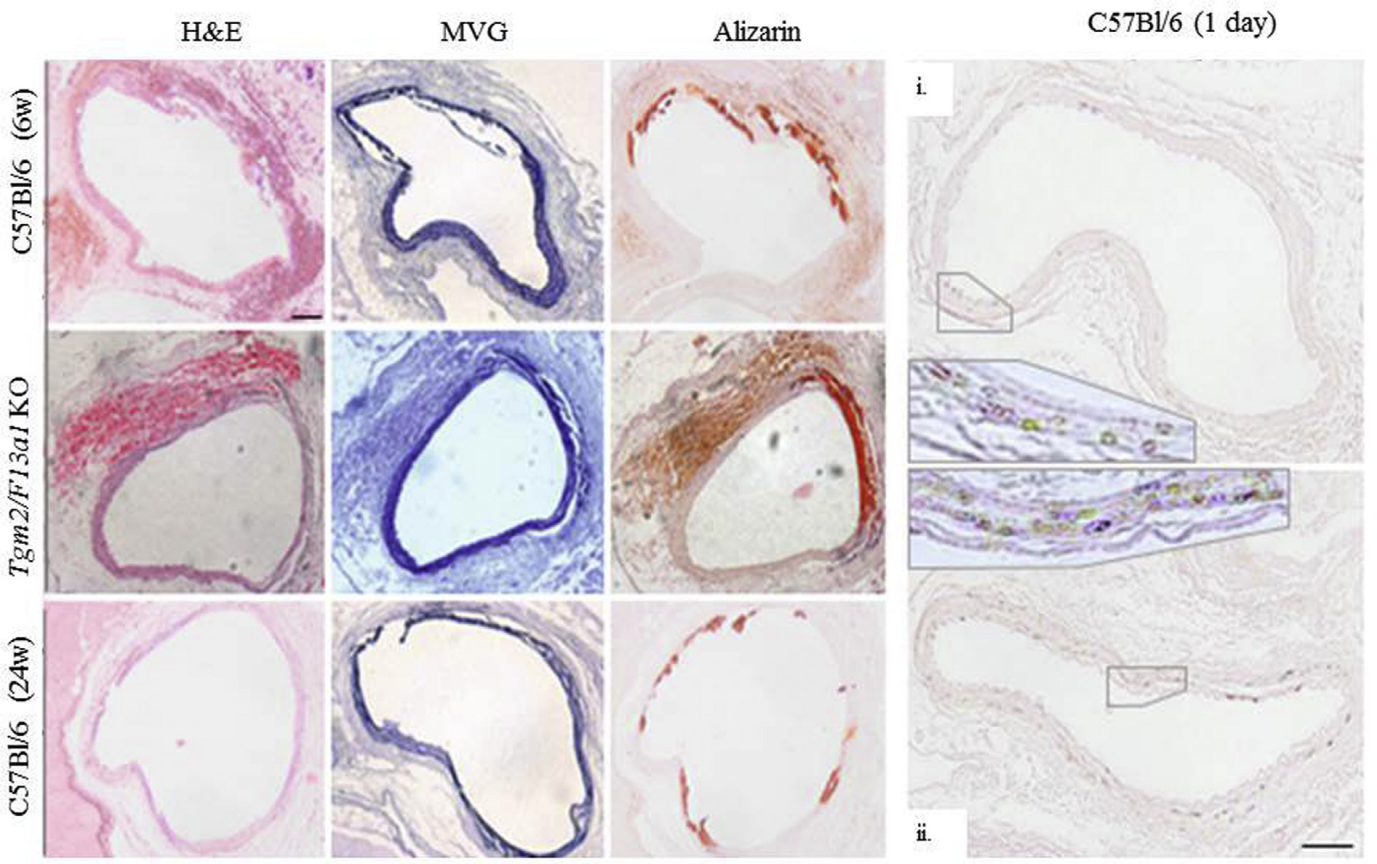
Medial degeneration is associated with calcification in treated mouse aortas. **A,** Sections of mouse aorta obtained 6 weeks or 24 weeks after treatment with CaCl_2_ and stained with hematoxylin and eosin (*H&E*), Miller's elastic Van Gieson (*MVG*) stain, and alizarin red S for deposition of calcium. Intense staining for calcium is apparent at the injured ventral face of the aorta in all genotypes 6 weeks after CaCl_2_ treatment. Representative images from wild-type and *Tgm2*^*–/–*^*/F13a1*^*–/–*^ double knockout (*KO*) mice are shown. Calcium deposition was also apparent 24 weeks after CaCl_2_ deposition, when in some mice, medial degeneration and calcification were apparent around the whole circumference of the vessel. **B,** Aorta from two representative C57Bl/6J wild-type mice examined 24 hours after CaCl_2_ treatment. Alizarin red S staining showed occasional small yellow orange Ca^2+^ containing crystals in the lamellae, but not widespread Ca^2+^ deposition, verifying that CaCl_2_ treatment did not immediately flood the aorta with Ca^2+^ ions, but initiated tissue damage.

Despite evidence that either FXIII-A or TG2 activity is necessary for ectopic calcification,^38^ fragmented elastin underwent calcification^39^ in all genotypes including *Tgm*^–/–^/*F13a1*^*–/–*^ mice. Calcification persisted for at least 24 weeks in wild-type (Fig 8, *A*) and *Tgm2*^*–/–*^ (not shown) mice.

### Gene expression studies revealed marked alterations in aneurysmal arteries from mice lacking FXIII-A

**Fig 9:**
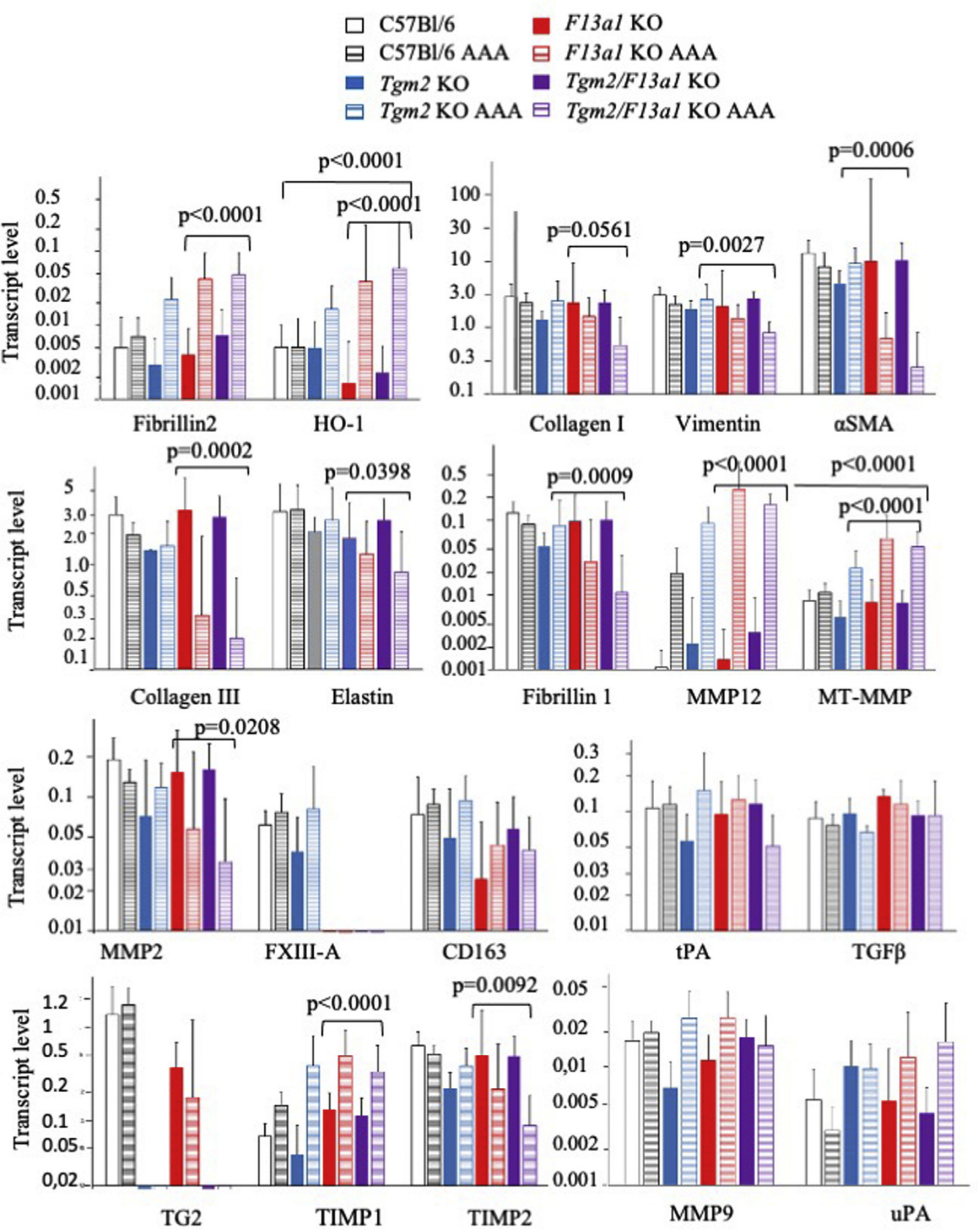
Transcript levels in aortas in control and aneurysmal aortas. Transcript levels in aortas collected 6 weeks after CaCl_2_ treatment were determined by reverse transcriptase polymerase chain reaction and normalized to the levels of ribosomal protein large subunit (Rpl)32. Mean values are shown with 95% confidence intervals. Data are presented on a logarithmic scale for clarity. Statistically significant increases upon aneurysm induction were observed in messenger RNAs (mRNAs) encoding heme oxygenase 1, matrix metalloproteinase 12 (*MMP12*) and membrane-type MMP (*MT-MMP*), comparing aneurysmal and nonoperated aortas mice from all genotypes. For other transcripts including CD163, tissue type plasminogen activator (*tPA*), transforming growth factor (*TGF*)-β, no significant changes in levels were observed between aneurysmal and non-operated aortas. Surprisingly marked decreases in mRNAs encoding α-smooth muscle actin (*α-SMA*) and collagen III were seen in both Tgm2^–/–^/F13a1^–/–^ mice and F13a1^–/–^ mice and significant decreases in mRNAs encoding elastin and MMP2 were also apparent in these genotypes. Probability values shown here compare aneurysmal and nonaneurysmal aortas from all mice deficient in factor XIII-A (*FXIII-A*). *HO-1,* Heme oxygenase 1; *TG,* Transglutaminase.

Because visual inspection could not easily determine the boundary between injured and uninjured tissue, RNA was isolated from the aorta extending from the aortic arch to the iliac bifurcation to ensure comparability between samples. Serial sectioning suggested that at least 25% of the excised tissue was injured (not shown). Apart from the targeted genes in knockout mice, transcript levels did not show consistent differences between genotypes. Normalized levels of transcripts including MMP12, MT1-MMP (MMP14), and heme oxygenase increased after aneurysm development, particularly in the gene knockout mice, consistent with the invasion of inflammatory macrophages (Fig 9). In contrast, transcripts encoding FXIII-A and CD163 (present in resident macrophages) did not show statistically significant changes in expression. Further, TG2 mRNA did not increase after aneurysm induction in either wild-type or *F13a1*^*–/–*^ mice (Fig 9).

Transcripts including vimentin, smooth muscle α-actin and collagen III (possibly also collagen I; *P* = .056) showed decreases in aneurysmal arteries from both *F13a1* knockout and *Tgm2/F13a1* double knockout mice relative to uninjured aortas from mice of the same genotypes. MMP2 mRNA was also decreased in injured relative to uninjured aortas in these mice, whereas MMP9 showed no significant difference in the levels between the genotypes. Fibrillin I mRNA levels decreased, whereas fibrillin 2 mRNA increased in FXIII-A-deficient mice, suggesting reversion to a more fetal pattern of gene expression^40^; there were also reciprocal changes between TIMP1 (expression increased in gene knockout mice) and TIMP2 (expression decreased) (Fig 9).

The mRNAs for TG1 and TG7 increased in each of the three mouse gene knockout lines (*P* < .01 in each case), but the low initial concentrations of these mRNAs, relative to the mRNAs encoding TG2 and FXIII-A (Fig 1), mean that it is unlikely that they had increased sufficiently to compensate for either knockout. Although TG3 was detected at appreciable levels in uninjured aorta, no statistically significant changes in TG3 mRNA were observed upon aneurysm induction (*P* > .7).

## Discussion

The risk that AAA poses to human health has prompted the development of several small animal models, including induction of AAA by periarterial application of CaCl_2_.^37^^,^^41^ Munezane et al^25^ reported that aneurysms of the rat infrarenal aorta induced by combined CaCl_2_ and luminal elastase treatment showed a marked rise in TG2 expression and suggested that this might constitute a protective response.

Here we have tested whether TG2 influences CaCl_2_-induced AAA development by comparing wild-type and *Tgm2*^*–/–*^ mice. *Tgm2*^*–/–*^ mice showed a marginal increase in dilatation relative to wild-type mice 6 weeks after CaCl_2_ treatment, but dilatation then continued from 6 to 24 weeks in the *Tgm2*^*–/–*^ but not wild-type mice, suggesting a role for TG2 in arterial protection or repair. Because FXIII-A might act redundantly with TG2 to mediate protection or repair, we also induced aneurysms in *Tgm2*^*–/–*^*/F13a1*^*–/–*^ and *F13a1*^*–/–*^ mice. Dilatation was unaltered in *F13a1* mice, whereas, in contrast with expectations, dilatation was decreased in *Tgm2*^*–/–*^*/F13a1*^*–/–*^ mice.

We cannot account for the decreased dilatation of aortas from *Tgm2*^*–/–*^*/F13a1*^*–/–*^ mice, although the basal tension and the elastin/collagen ratio of their aortas appeared different from other genotypes. *Tgm2*^*–/–*^*/F13a1*^*–/–*^ mice also develop red blood cell extravasation and myocardial fibrosis,^26^ whereas altered fibronectin metabolism was noted in a separate cohort of *Tgm2*^*–/–*^*/F13a1*^*–/–*^ mice.^28^ It is unclear whether common factors underlie the fibrosis, alterations in fibronectin metabolism, and the arterial changes seen here. Regardless of this finding, our results show that FXIII-A does not act redundantly with TG2 to protect against aneurysm development in the CaCl_2_ model.

Human aneurysms frequently exhibit a decreased elastin/collagen ratio, owing to preferential degradation of elastin coupled, in some instances, with increased collagen deposition.^27^^,^^42^ We observed lower periaortic collagen density adjacent to the injured face of mouse aneurysms, both at 6 and 24 weeks after injury, suggesting that compensatory collagen deposition had not occurred. Periaortic collagen density was also lower at 24 weeks in *Tgm2*^*–/–*^ mice than in wild-type mice, corresponding with the increased dilatation in these animals. A decrease in TG2-mediated cross-linking and consequent increase in susceptibility to proteolysis could explain the decreased density of collagen in the *Tgm2*^–/–^ mice, although verifying this finding by quantifying isopeptide cross-links remains difficult.^43^^,^^44^ Alternatively, the absence of intracellular TG2 may have altered cellular function^13^ and hence the deposition of collagen.

Although we observed calcification of mouse AAA, rat AAA did not calcify in the study of Munezane et al^25^ and this difference in aneurysm morphology was accompanied by differences in gene expression profiles. In particular, we did not observe increased levels of mRNAs encoding TG2, MMP2, or MMP9,^25^ although similar to Longo et al^45^ we observed an increase in MMP12 mRNA. Our results indicate that an increase in TG2 mRNA levels is not an invariable response to vessel dilatation, and similarly using a microarray approach, Biros et al^46^ found that TG2 mRNA did not increase within human AAA, but that it did increase in aortic obstructive disease. We have stained AAA sections from four patients with a polyclonal antibody to TG2 and have observed intense areas of striated staining in isolated areas of inflammation, but also large areas devoid of staining (not shown). Therefore, localized expression of TG2 could be important in the response to disease, even if an average increase in mRNA expression across the whole lesion is not evident

A proportion of mice that lacked FXIII-A died within a few days of laparotomy. Necropsy frequently revealed cardiac pathology (eg, atrial thrombosis) in C57Bl/6J *F13a1*^*–/–*^ mice, sometimes associated with distal ischemia, and with clots that may have embolized from the heart. In addition to this pathology, *Tgm2*^*–/–*^*/F13a1*^*–/–*^ mice showed an increased frequency of hemorrhage relative to *F13a1*^*–/–*^ mice, that may have resulted from delayed bleeding caused by surgical injury or postoperative stress. It seems that compromised tissue protection and repair in the absence of TG2 exposes the bleeding diathesis associated with FXIII-A deficiency, similar to the situation where TG2 deficiency exacerbates extravasation and myocardial fibrosis associated with FXIII-A deficiency.^26^ The cause of the nonhemorrhagic deaths is uncertain, but FXIII-A also maintains endothelial barrier function^26^^,^^47^ and can protect against systemic organ injury.^48^^,^^49^ Impaired barrier function may have rendered *F13a1* knockout mice susceptible to cardiac damage after laparotomy. We also observed large decreases in the mRNAs encoding certain structural proteins after aneurysm induction, particularly in mice lacking FXIII-A and note that Kothapalli et al^50^ observed decreases of similar magnitude in collagen and elastin expression in cells explanted from CaCl_2_-induced rat aortic aneurysms, although presumably these cells expressed FXIII-A. Further work is needed to determine whether common pathways underlie the unexpected changes in gene expression in aneurysmal aortas from *F13a1*^–/–^ knockout mice and the changes in endothelial barrier function, and hence mouse survival.

Of the three models commonly used to induce AAA in mice, the relevance of the angiotensin II-hyperlipidemia protocol has been questioned.^51^ The infusion of elastase into the aortic lumen causes rapid dilatation, but would risk hemorrhage in mice lacking FXIII-A. Further, ligation necessary for elastase infusion is expected to preferentially damage *Tgm2*^*–/–*^ vessels,^26^ complicating the analysis of aneurysm development. We therefore chose the CaCl_2_ protocol to minimize manipulation of the aorta. A limitation of the CaCl_2_ model is that murine aneurysms do not accumulate luminal thrombus, possibly because of rapid fibrinolysis.^52^^,^^53^ Second, CaCl_2_-induced aneurysms rarely rupture and cannot model protection against this outcome. Third, mice do not express proelafin/trappin-2, a neutrophil elastase inhibitor, which is cross-linked by TG(s) to the extracellular matrix^54^ and which may confer protection in human arterial lesions.^17^

A point of difference between the C57BL/6J mice used here and the mixed strain mice used previously is that ligation caused carotid artery rupture in 50% of mixed strain *apoE*^*–/–*^*/Tgm2*^*–/–*^*/F13a1*^*–/–*^ triple knockout mice and elastic breakage without rupture in *apoE*^*–/–*^*/Tgm2*^*–/–*^ mice,^26^ whereas ligation did not cause carotid rupture in either C57BL/6J *Tgm2*^*–/–*^*/F13a1*^*–/–*^ or C57BL/6J *apoE*^*–/–*^*/Tgm2*^*–/–*^*/F13a1*^*–/–*^ mice (n = 10). Nevertheless, ligation induced the deposition of additional elastic lamellae in C57BL/6J mice lacking TG2 (not shown), presumably to reinforce the intrinsically weaker vessel against mechanical stress, and showing that the elastase protocol might prove problematic in these mice. We suspect that rupture depended on the additional stress caused by neointimal deposition, which was occlusive in the mixed strain mice, but, as reported,^55^^,^^56^ was minimal or absent in C57BL/6J mice.

Regardless of these caveats, we conclude that the basal level of TG2 expression in aorta appears sufficient to limit mouse AAA development. We did not detect induction of TG2 mRNA expression upon injury, but do not exclude the possibility that localized increases in TG2 expression may have occurred but were not apparent in total tissue measurements. Experiments were not done that would confirm that the replacement or enhancement of TG2 would be protective; however, because TG2 expression is one of a limited number of enzymes implicated in tissue repair (and its expression can be induced pharmacologically^10^), further studies are merited to determine whether TG2 may prove a future viable target for the management of human AAA.

## Author contributions

Conception and design: KG, CJ, DS, RP

Analysis and interpretation: KG, LN, SI, CJ, RP

Data collection: KG, KS, CB, LC, NY

Writing the article: KG, RP

Critical revision of the article: KS, CB, LN, LC, NY, SI, CJ, DS

Final approval of the article: KG, KS, CB, LN, LC, NY, SI, CJ, DS, RP

Statistical analysis: KG, KS, CB

Obtained funding: KG, DS, RP

Overall responsibility: RP

## Figures and Tables

**Supplementary Table (online only):** Primer sequences for reverse transcriptase polymerase chain reaction

Gene	Forward primer	Reverse primer
*Elastin*	AAAGCCTGGGAAAGTTCCTG	TACACCTGGAAGACCAACAC
*Col I*	ATGGATTCCCGTTCGAGTACG	TCAGCTGGATAGCGACATCG
*Col III*	CACCCTTCTTCATCCCACTCTTA	ACCAAGGTGGCTGCATCC
*Vimentin*	CGGAAAGTGGAATCCTTGCA	CACATCGATCTGGACATGGCTGT
*aSMA*	ACTGGGACGACATGGAAAAG	GTTCAGTGGTGCCTCTGTCA
*MMP2*	CTGATAACCTGGATGCCGTCGT	TGCTTCCAAACTTCACGCTCTT
*MMP9*	GTCTCGGGAAGGCTCTGCTGTT	CTCTGGGGATCCACCTTCTGAG
*FXIII-A*	TGCTGGTGTCTTTAACACATTTTTAA	TGGGCCGAGAATGAATTGGT
*TG1*	TTCGCTACCCGTACCGTCA	CTTCATCCAGCAGTCGTT
*TG2*	ATTGGCAGRGRGGACATTC	TCGTGGGCGGAGTTGTA
*TG3*	AAGAAGCTGACCATGAGTGCTTT	TGCGCCCTTCGATTCATAG
*TG4*	CCCATCTATTTGACCATAACTTGAA	GCGAGAAACACCCTTGATT
*TG5*	AGGCAGGATTCTGGAGAATATG	GGGCCACAGCCACAGCAGTAGAG
*TG6*	TCCGAGTCAAT GTGAGCG	GTCTTCTGTCAGGTCTCCTTTGTA
*TG7*	ATGTGCACGGTAATGAGATGCT	TGTGTGCAGAATGGAAATTGG
*CD163*	ATGGGTGGACACAGAATGGTT	CAGGAGCGTTAGTGACAGCAG
*tPA*	CAACAGCGGCCTGGTACAA	CCCCATTGAAGCATCTTGGTT
*uPA*	GAAACCCTACAATGCCCACAGA	GACAAACTGCCTTAGGCCAATC
*PAI-1*	ACGGTGATGCGATATAATGTAAACG	CATTCCTGAGAACACAGCATTG
*Fibrillin 1*	CCTGTGCTATGATGGGTTCA	AGGTCCCACTAAGGCAGATG
*Fibrillin 2*	CCACTCCTATTGCTGCCCAG	TTGGGGCGGGAACAGAATC
*MT-MMP*	CCCAAGGCAGCAACTTCA	CAATGGCAGCTGAGAGTGAC
*HO-1*	AACAAGCAGAACCCAGTCTATG	TGAGCAGGAAGGCGGTCTTA
*MMP12*	GCTAGAAGCAACTGGGCAAC	ACCGCTTCATCCATCTTGAC
*TIMP1*	GTGGGAAATGCCGCAGCAGAT	GGGCATATCCACAGAGGCTTT
*TIMP2*	CCAGAAGAAGAGCCTGAACCA	GTCCATCCAGAGGCACTCATC
*TGFB*	CACCGGAGAGCCCTGGATA	TGTACAGCTGCCGCACACA
*B-Actin*	CGTGAAAAGATGACCCAGATCA	TCGTACGACCAGAGGCATACAG
*RPL32*	AAAATTAAGCGAAACTGGCGG	TGTTGCTCCCATAACCGATG

Except for mouse factor XIII-A, primers to mouse transglutaminases were as described by Johnson et al.^1^ Mouse factor XIII-A primers spanning the boundary of coding exons 7 and 8 (mRNA exons 8 and 9), were designed using Primer Express. Other primers were obtained from primer bank (http://pga.mgh.harvard.edu/primerbank/).

## Appendix

Additional material for this article may be found online at www.jvsvenous.org.
